# Tiny capillary hemangioma of the ileum as the cause of chronic anemia

**DOI:** 10.1002/jgh3.12378

**Published:** 2020-06-22

**Authors:** Naoto Iwai, Takashi Okuda, Kohei Oka, Tasuku Hara, Yutaka Inada, Toshifumi Tsuji, Toshiyuki Komaki, Yoshito Itoh, Keizo Kagawa

**Affiliations:** ^1^ Department of Gastroenterology and Hepatology Fukuchiyama City Hospital Kyoto Japan; ^2^ Department of Molecular Gastroenterology and Hepatology Graduate School of Medical Science, Kyoto Prefectural University of Medicine Kyoto Japan

**Keywords:** anemia, capillary hemangioma, ileum

## Abstract

A 70‐year‐old male was diagnosed with tiny capillary hemangioma of the ileum using double‐balloon endoscopy. Capillary hemangiomas should be included in the differential diagnoses of OGIB.

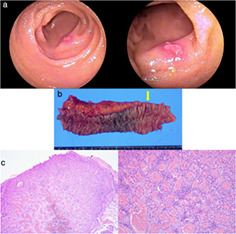

For two consecutive years, a 70‐year‐old male patient presented with persistent iron deficiency anemia. Laboratory investigations showed a hemoglobin level of 9.9 g/dL, hematocrit of 35.8%, and mean corpuscular volume of 73.2 fL. Physical examination showed no signs of vascular changes in the skin. Investigations such as contrast‐enhanced computed tomography, esophagogastroduodenoscopy, and colonoscopy could not determine the source of the patient's bleeding. Transoral double‐balloon endoscopy revealed no lesions; however, transanal double‐balloon endoscopy revealed a submucosal‐like lesion with ulceration in the ileum (Fig. 1a). As the lesion showed marked redness without active bleeding on endoscopy, it was suspected to be the cause of the bleeding. Endoscopic treatment might have resulted in incomplete resection because of the submucosal‐like appearance. Thereafter, endoscopic tattooing with India ink was performed to clarify the location of the lesion during surgical resection. Subsequently, a video capsule endoscopy throughout the small intestine identified the solitary lesion accompanied by bleeding without any other lesions. The patient underwent laparoscopic enterectomy. Intraoperatively, the lesion showed no signs of mesenteric involvement and was identified in the ileum 70 cm proximal to the terminal ileum based on the India ink tattoo. Macroscopic examination of the resected specimen showed a raised lesion (8 mm in diameter) with an ulcer (Fig. 1b). In contrast, microscopic examination revealed proliferation of the capillary vessels, suggesting the presence of a capillary hemangioma (Fig. 1c). Postoperatively, the clinical course was uneventful, and his anemia improved. A month after the enterectomy, laboratory investigation revealed hemoglobin levels of 13.9 g/dL, hematocrit of 40.8%, and mean corpuscular volume of 89.5 fL.

**Figure 1:**
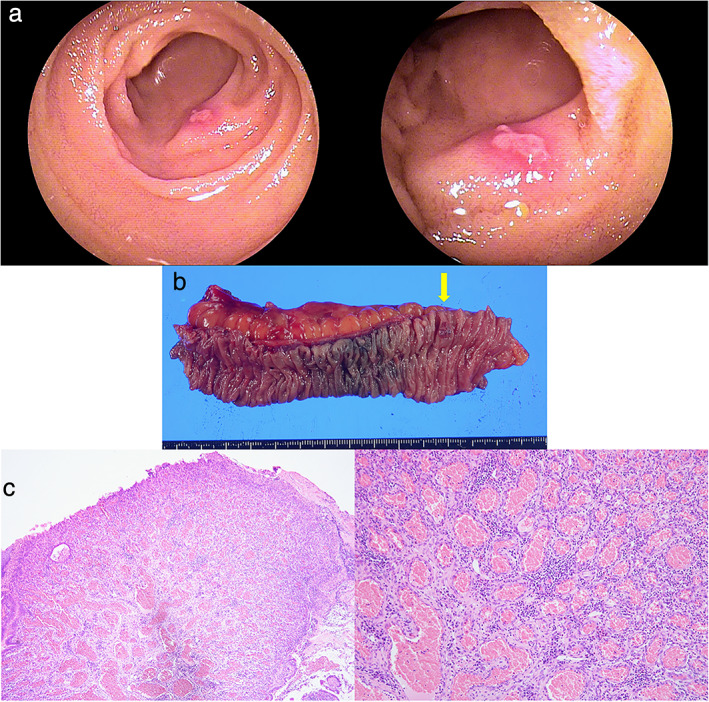
(a) A transanal double‐balloon endoscopy reveals a submucosal‐like lesion with ulceration in the ileum. (b) Macroscopic examination of the resected specimen shows a raised lesion 8 mm in diameter with an ulcer (yellow arrow). (c) Microscopic examination shows proliferation of capillary vessels.

Hemangioma of the small intestine is a rare benign vascular tumor, which causes obscure gastrointestinal bleeding (OGIB).^1^ Hemangiomas of the small intestine are classified as cavernous hemangiomas, capillary hemangiomas, and hemangiomas associated with multisystem vascular diseases. Capillary hemangiomas are defined as solitary lesions <20 mm in size.^2^ With the prevalence of capsule endoscopy and balloon‐assisted enteroscopy, preoperative diagnosis of small intestinal hemangioma has become reliable.^3^ In the present study, the ulcer in the capillary hemangioma was proposed to be the cause of occult bleeding and consequent chronic iron deficiency anemia. Endoscopic treatment, such as endoscopic resection, injection of sclerosant, and argon plasma coagulation, may be an effective therapeutic strategy for capillary hemangioma. However, endoscopic treatment may result in poor therapeutic outcomes because the main lesion is generally located in the submucosal layer.^3^ Considering that the lesion showed a submucosal‐like tumor with ulceration, surgical resection was performed as opposed to endoscopic treatment. To conclude, capillary hemangiomas should be included in the differential diagnoses of OGIB.
